# The gene–treatment interaction of paraoxonase-1 gene polymorphism and statin therapy on insulin secretion in Japanese patients with type 2 diabetes: Fukuoka diabetes registry

**DOI:** 10.1186/s12881-017-0509-1

**Published:** 2017-12-12

**Authors:** Akiko Sumi, Udai Nakamura, Masanori Iwase, Hiroki Fujii, Toshiaki Ohkuma, Hitoshi Ide, Tamaki Jodai-Kitamura, Yuji Komorita, Masahito Yoshinari, Yoichiro Hirakawa, Atsushi Hirano, Michiaki Kubo, Takanari Kitazono

**Affiliations:** 1 Department of Medicine and Clinical Science, Graduate School of Medical Sciences, Kyushu University, 3-1-1 Maidashi, Higashi-ku, Fukuoka, 812-8582 Japan; 2 Diabetes Center, Hakujyuji Hospital, Fukuoka, Japan; 3 Centre for Cohort Studies, Graduate School of Medical Sciences Kyushu University, Fukuoka, Japan; 4 The George Institute for Global Health University of Sydney, Sydney, Australia; 5 Division of General Internal Medicine, School of Oral Health Science, Kyushu Dental University, Kitakyushu, Japan; 6 Epidemiology and Public Health, Graduate School of Medical Sciences, Kyushu University, Fukuoka, Japan; 7 Center for Integrative Medical Sciences, RIKEN, Yokohama, Japan

**Keywords:** Gene–treatment interaction, *PON1* Q192R polymorphism, Statin therapy, Insulin secretion

## Abstract

**Background:**

Although statins deteriorate glucose metabolism, their glucose-lowering effects have emerged in some situations. Here, we assessed whether these effects are a consequence of statins’ interaction with paraoxonase (PON)1 enzyme polymorphism.

**Methods:**

Adult Japanese type 2 diabetes patients (*n* = 3798) were enrolled in a cross-sectional study. We used Q192R polymorphism of the *PON1* gene as a representative single-nucleotide polymorphism and focused on the effects of the wild-type Q allele, in an additive manner. For patients with and without statin therapy, the associations of this allele with fasting plasma glucose (FPG), HbA_1c_, C-peptide, HOMA2-%β, and HOMA2-IR were investigated separately using a linear regression model, and were compared between groups by testing interactions. Sensitivity analyses were performed using propensity score to further control the imbalance of characteristics between groups.

**Results:**

Among patients with statin therapy, there were linear associations of the number of Q alleles with decreased FPG and HbA_1c_, and with increased serum C peptide and HOMA2-%β (all *P* < 0.01 for trends), while such associations were not observed among those without statin therapy. These differences were statistically significant only for serum C peptide and HOMA2-%β (*P* < 0.01 for interactions). These associations remained significant after multiple explanatory variable adjustment. Sensitivity analyses using propensity score showed broad consistency of these associations.

**Conclusions:**

Patients with the Q allele of the *PON1* Q192R polymorphism who were treated with statins exhibited improvement in glucose metabolism, especially in insulin secretion, suggesting the importance of genotyping *PON1* Q192R to identify those who could benefit from statin therapy.

**Electronic supplementary material:**

The online version of this article (10.1186/s12881-017-0509-1) contains supplementary material, which is available to authorized users.

## Background

Hypercholesterolemia is a common comorbidity with diabetes and contributes to the increased risk of cardiovascular disease among affected people [1]. Lipid-lowering with 3-hydroxy-3-methylglutaryl-coenzyme A reductase inhibitors (statins) is an important and recommended therapy to reduce cardiovascular risk and to treat atherosclerosis associated with hypercholesterolemia [1]. Recently, however, several studies have reported adverse effects of statins on glucose metabolism because statins reduce both insulin secretion and insulin sensitivity, and, as a result, deteriorate glycemic control [2–5], although glucose-lowering effects of statins have emerged in some situations [6]. For the improved management of diabetes and lipids, there is a need for a deeper understanding of the glucose-raising effects of statins and the ability to prevent such effects.

Gene–treatment/environment interaction analysis investigates whether the magnitude of the genetic effect estimate differs across the range of treatments or environmental factors [7], and could contribute to revealing the pharmacological mechanism of the enzyme by investigating the association of common genetic variants in the gene encoding that enzyme and treatment factors. 3-Hydroxy-3-methylglutaryl-coenzyme A reductase (HMGCR) is the main enzyme inhibited by statins; its genetic variants have been shown to be associated with increased body weight and the risk of type 2 diabetes among nondiabetic subjects, independently of statin therapy [8]. Paraoxonase (PON) 1 is another enzyme potentially affected by statins [9], which possesses the properties of an antioxidant and an insulin secretagogue [10, 11]. Since the glucose-lowering effects of statins are most pronounced in patients with an improvement in HDL-C upon statin therapy [6] and this improvement was shown to be dependent on the genotype of *PON1* [12], it could be hypothesized that the interaction of the *PON1* genotype with statins could play a role in changing glucose metabolism in patients treated with statins. However, at present, there is a lack of evidence for such an interaction in patients with type 2 diabetes.

Therefore, in the present study, we performed a quantitative trait interaction analysis testing modifiable effects of statins on the association between *PON1* Q192R polymorphism and glycemia, such as fasting plasma glucose, HbA_1c,_ insulin secretion measured by serum C-peptide and HOMA2-%β, and insulin resistance measured by HOMA2-IR, in Japanese patients with type 2 diabetes.

## Methods

### Study participants

The Fukuoka Diabetes Registry is a multicenter, prospective study designed to investigate the influence of modern therapy on the prognosis of patients with diabetes mellitus in Japan. Patients who regularly attended teaching hospitals authorized by the Japan Diabetes Society or certified diabetes clinics in Fukuoka Prefecture (UMIN Clinical Trial Registry 000002627) [13] were registered between April 2008 and October 2010 if aged ≥20 years. Exclusion criteria were as follows: 1) patients with drug-induced diabetes mellitus or receiving corticosteroid therapy; 2) patients who had undergone renal replacement therapy; 3) patients with serious diseases other than diabetes, such as advanced malignancy or decompensated liver cirrhosis; and 4) patients unable to visit diabetologists regularly. Among the 5131 patients registered, after excluding those with type 1 diabetes defined by serum C-peptide level < 0.03 nmol/l and being on insulin therapy, those who had already eaten breakfast, those with unacceptable levels of plasma glucose (<3 mmol/l or >25 mmol/l) or C-peptide (<0.2 nmol/l or >3.5 nmol/l) for HOMA2-%β [14], and those who had not been genotyped for the *PON1* gene, the remaining 3798 patients were enrolled in the present cross-sectional study. The present study was conducted with the approval of Kyushu University Institutional Review Board, and written informed consent was obtained from all of the participants.

### Clinical evaluation and laboratory measurements

The participants completed a self-administered questionnaire to provide data on their smoking habits, duration of diabetes mellitus, alcohol intake, physical activity, family history of diabetes, and past history of cardiovascular disease. Smoking habits and alcohol intake were classified as either current use or not. Metabolic equivalent (MET) hours per week values were calculated using Ainsworth’s methods [15]. The participants’ medical records were reviewed for all medications, including statin therapy, oral hypoglycemic agents (OHA), and insulin therapy. Body weight and height were measured, and body mass index (BMI) was calculated as weight (kg) divided by height squared (m^2^). Blood pressure was measured with the participants in a sitting position. Hypertension was defined as blood pressure ≥ 140/90 mmHg and/or current use of antihypertensive agents. Blood samples were collected via venipuncture under fasting conditions. Hemoglobin A_1c_ (HbA_1c_) level was determined by high-performance liquid chromatography (Tosoh Corp., Tokyo, Japan), plasma glucose by the glucose oxidase method, serum C-peptide by a chemiluminescent immunoassay (Kyowa Medex, Tokyo, Japan), and lipid profiles, such as serum total cholesterol, LDL-C, HDL-C, and triglyceride, by enzymatic methods. Beta-cell function and insulin resistance were estimated based on fasting glucose and C-peptide concentrations using the homeostasis model assessment (HOMA) calculator, version 2.2.2 (http://www.dtu.ox.ac.uk, accessed June 2012), and are expressed as the homeostasis model assessment of β-cell function (HOMA2-%β) and the homeostasis model assessment of insulin resistance (HOMA2-IR), respectively.

### Genotyping of PON1 Q192R polymorphism

The gene encoding the paraoxonase (PON) 1 enzyme has a polymorphism with a reference single-nucleotide polymorphism (SNP) ID number of rs662; this SNP changes amino acid 192 of the PON1 protein. The wild-type, rs662(A), encodes a glutamine (Q), while the variant, rs662(G), encodes an arginine (R).The genotypes of the *PON1* Q192R SNP were determined as QQ, QR, or RR using multiplex polymerase chain reaction-based invader assays (Third Wave Technologies, Madison, WI, USA) [16]. In the present analyses, we focused on the effect of the Q-allele, the wild type, compared with the R-allele, as the former confers the high antioxidative effect of the enzyme [9].

### Statistical analysis

The data on C-peptide, HOMA2-%β, HOMA2-IR, and triglyceride were log-transformed for the statistical analysis due to their skewed distribution. The chi-square test was used to test the divergence from Hardy–Weinberg equilibrium by determining the difference between observed and expected genotype frequencies from the allele frequencies. The clinical and biochemical characteristics of the participants were compared between those using and not using statins, and between *PON1* Q192R genotypes using analysis of variance (ANOVA), chi-square analyses, and Fisher’s exact tests.

To investigate a gene–treatment interaction on glycemic parameters, the associations of *PON1* Q192R genotypes with fasting plasma glucose (FPG), HbA_1c_, C-peptide, HOMA2-%β, and HOMA2-IR were estimated separately using linear regression models in patients with and without statin therapy, wherein the effects of *PON1* Q192R genotypes were assumed to be additive, namely, the number of Q alleles was counted. These associations were compared using the interaction term of genotypes and use of statins in the relevant statistical model. In multiple explanatory variable analyses, adjustments were made for gender, age, BMI, HbA_1c_, OHA, insulin therapy, current smoking, current drinking, leisure-time physical activity, and duration of diabetes.

In a nonrandomized study, patients with specific medication might be at higher risk than those without, since there could be a bias in administering medication to severe patients. Such imbalance of background risk might affect the results. Therefore, we performed sensitivity analyses using propensity score (PS) to further control the difference of characteristics between patients with and without statins. The PS for the probability of receiving statins was estimated by a multiple explanatory variable logistic regression model using the following clinical variables that might influence the intention to prescribe statins: gender; age; BMI; current smoking and drinking; duration of diabetes; hypertension; past history of stroke, ischemic heart disease, and arteriosclerosis obliterans; antiplatelet therapy; family history of diabetes and hyperlipidemia; leisure-time physical activity; FPG; HbA_1c_; LDL-C; HDL-C; logarithm of triglyceride; OHA (sulfonylurea, glinide, DPP4-I, α-GI, biguanide, thiazolidine); insulin therapy; fibrate therapy; ezetimibe therapy; ethyl eicosapentate therapy; and the method for controlling blood glucose (diet, OHA, insulin, combination of OHA and insulin). We performed three PS application analyses: one adjusting for PS, one matching patients with and without statin therapy using PS (1:1), and one using the inverse probability of treatment weights (IPTW). Matching for PS was performed in accordance with an optimization protocol using an SAS macro (PSMatch_Multi macro in SAS 9.4) [17], whereby cases and controls were matched if the difference of their scores was equal to or less than 0.01. All analyses were performed using the SAS software package version 9.3 (SAS Institute Inc., Cary, NC, USA). Values of *P* < 0.05 were considered to be statistically significant in all analyses.

## Results

Overall, 3798 patients were enrolled in the present study (Additional file 1: Table S1). Their mean age was 65 years and about half of the patients were male. Mean duration of diabetes was 14.6 years. Among the patients, 1678 were being treated with statins (atorvastatin 27.4%, pravastatin 26.1%, pitavastatin 19.6%, rosuvastatin 17.5%, simvastatin 6.6%, fluvastatin 2.8%), and they were older, with a longer duration of diabetes, higher values of HbA_1c_, and a greater frequency of being on oral hypoglycemic therapy than those who were not being treated with statins. The genotype proportions were 0.112 for QQ, 0.436 for QR, and 0.452 for RR (Additional file 2: Table S2), which indicated no divergence from Hardy–Weinberg equilibrium (*P* = 0.33). The distributions of genotypes were similar tendency in patients with and without statin therapy (*P* = 0.055 for the overall difference) (Table 1). Among all patients (Additional file 2: Table S2) and patients with and without statins (Table 1), the clinical and biochemical characteristics were almost all similar among the *PON1* genotypes, except for the rates of use of OHA and sulfonylurea.

**Table 1:** Clinical characteristics in statin-treated or untreated patients with type 2 diabetes mellitus according to PON1 genotype

	Statin (−)				Statin (+)			
PON1 Q192R genotype	QQ	QR	RR	*P* value	QQ	QR	RR	*P* value
	*N* = 249, 11.7%	*N* = 887, 41.8%	*N* = 984, 46.4%		*N* = 179, 10.7%	*N* = 767, 45.7%	*N* = 732, 43.6%	
Male, n (%)	167 (67.1)	573 (64.6)	648 (65.9)	0.73	78 (43.6)	371 (48.4)	333 (45.5)	0.37
Age, years	65.2 ± 10.6	64.8 ± 10.8	64.7 ± 10.6	0.83	66.1 ± 9.6	66.1 ± 9.4	65.9 ± 9.7	0.91
BMI, kg/m^2^	23.8 ± 3.5	23.8 ± 3.5	24.0 ± 3.9	0.28	24.5 ± 4.1	24.3 ± 3.8	24.4 ± 3.5	0.84
Duration of diabetes, years	13.8 ± 10.7	14.4 ± 10.2	14.4 ± 10.3	0.68	14.3 ± 10.1	14.6 ± 9.7	15.4 ± 9.9	0.17
Current smoker, n (%)	52 (20.9)	182 (20.5)	212 (21.5)	0.86	27 (15.1)	96 (12.5)	113 (15.4)	0.24
Current drinker, n (%)	118 (47.4)	398 (44.9)	430 (43.7)	0.57	57 (31.8)	244 (31.8)	247 (33.7)	0.71
Leisure-time physical activity, METs·h/w	18.9 ± 17.5	19.2 ± 19.2	18.6 ± 17.9	0.81	16.4 ± 18.5	18.4 ± 17.5	18.9 ± 18.7	0.25
Family history of diabetes, n (%)	132 (53.0)	491 (55.4)	525 (53.4)	0.64	98 (54.8)	446 (58.2)	400 (54.6)	0.36
Past history of CVD, n (%)	47 (18.9)	169 (19.1)	187 (19.0)	1.00	58 (32.4)	232 (30.3)	223 (30.5)	0.85
HDL cholesterol, mmol/l	1.41 ± 0.37	1.43 ± 0.37	1.41 ± 0.38	0.55	1.49 ± 0.36	1.46 ± 0.38	1.45 ± 0.36	0.40
LDL cholesterol, mmol/l	2.99 ± 0.75	3.03 ± 0.71	3.02 ± 0.75	0.77	2.75 ± 0.68	2.70 ± 0.61	2.69 ± 0.62	0.52
OHA, n (%)	144 (57.8)	587 (66.2)	645 (65.6)	0.043	124 (69.3)	540 (70.4)	525 (71.7)	0.76
Sulfonylurea, n (%)	91 (36.6)	397 (44.8)	423 (43.0)	0.07	77 (43.0)	390 (50.9)	362 (49.5)	0.17
Biguanide, n (%)	69 (27.7)	291 (32.8)	346 (35.2)	0.08	73 (40.8)	287 (37.4)	291 (39.8)	0.55
α-GI, n (%)	32 (12.9)	105 (11.8)	91 (9.3)	0.10	25 (14.0)	109 (14.2)	91 (12.4)	0.58
Thiazolidine, n (%)	20 (8.0)	111 (12.5)	114 (11.6)	0.15	36 (20.1)	148 (19.3)	112 (15.3)	0.08
Glinide, n (%)	16 (6.4)	60 (6.8)	58 (5.9)	0.74	7 (3.9)	47 (6.1)	41 (5.6)	0.51
DPP4-I, n (%)	0 (0)	4 (0.5)	6 (0.6)	0.45	1 (0.56)	2 (0.26)	2 (0.27)	0.64
Insulin, n (%)	57 (22.9)	178 (20.1)	200 (20.3)	0.61	39 (21.8)	145 (18.9)	154 (21.0)	0.50

Data are expressed as mean ± SD and n (percentage)

*CVD* cardiovascular disease, *OHA* oral hypoglycemic agents, *α-GI* alpha-glucosidase inhibitor, *DPP4-I* inhibitors of type 4 dipeptidyl peptidase

**Fig. 1:**
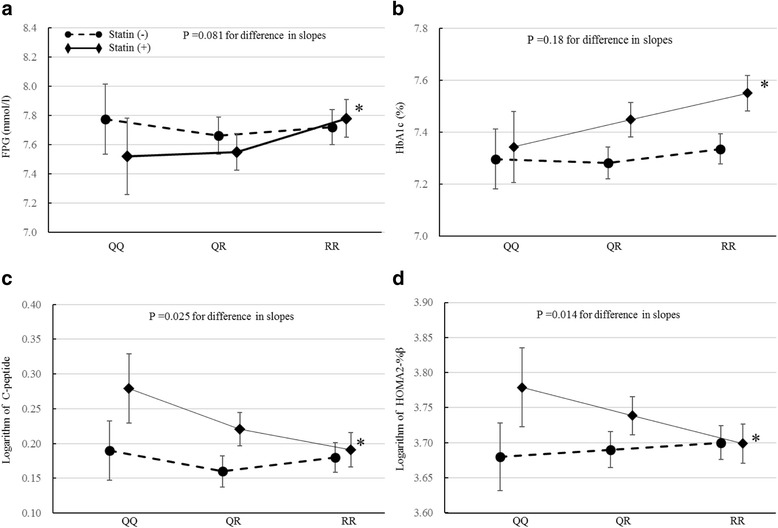
Association of PON1 Q192R polymorphism with FPG (**a**), HbA1c (**b**), serum C-peptide (**c**), and HOMA2-%β (**d**) after stratified analysis by statin therapy. Data are expressed as adjusted mean (95% CI) referring to ANCOVA; *P* value refers to multiple regression analysis. FPG and HbA_1c_: adjusted for gender, age, BMI, smoking, alcohol, leisure-time physical activity, duration of diabetes, SU, glinide, thiazolidine, α-GI, biguanide, insulin. C-peptide and HOMA2-%β: adjusted for gender, age, BMI, smoking, alcohol, leisure-time physical activity, duration of diabetes, HbA_1c_, SU, glinide, thiazolidine, α-GI, biguanide, insulin. * *P* < 0.05 for trend in the group with statins

In the patients with statin therapy, the number of Q alleles was associated with decreased values of FPG and HbA_1c_, and with increased C-peptide and HOMA2-%β (*P* = 0.0052, 0.0022, 0.016, and 0.0001 for the trends, respectively), and there was a tendency for a positive association with HOMA2-IR (*P* = 0.082 for trend) (Table 2). In patients without statin therapy, however, no such associations of the number of Q alleles with any of the glycemic parameters were observed. The difference in such associations between those with and without statins was significant for C-peptide and HOMA2-%β, and was marginally insignificant for FPG and HOMA2-IR (Table 2). In the multiple explanatory variable analyses (Fig. 1), there was consistency of these associations, but the heterogeneity in the association of Q alleles with C-peptide and HOMA2-%β remained statistically significant.

**Table 2:** Association of PON1 Q192R polymorphism with glycemia, insulin secretion, and sensitivity after stratified analysis by statin therapy

		FPG, mmol/l	HbA_1c_, % (mmol/mol)	Logarithm of C-peptide	Logarithm of HOMA2-%β	Logarithm of HOMA2-IR
Statin (−)	QQ	7.75 ± 2.02	7.23 ± 0.95 (55.5 ± 10.4)	0.19 ± 0.39	3.69 ± 0.45	0.02 ± 0.43
	QR	7.66 ± 2.04	7.28 ± 1.03 (56.1 ± 11.2)	0.16 ± 0.38	3.69 ± 0.48	−0.02 ± 0.41
	RR	7.73 ± 2.06	7.35 ± 1.05 (56.8 ± 11.5)	0.19 ± 0.39	3.69 ± 0.50	0.01 ± 0.41
	*P* value	0.82	0.07	0.47	0.8	0.47
Statin (+)	QQ	7.51 ± 1.79	7.34 ± 0.92 (56.7 ± 10.1)^#^	0.27 ± 0.40^#^	3.80 ± 0.49^#^	0.09 ± 0.41
	QR	7.53 ± 1.88^#^	7.43 ± 0.99 (57.7 ± 10.8)	0.22 ± 0.38	3.76 ± 0.47^#^	0.04 ± 0.40
	RR	7.81 ± 1.98	7.56 ± 1.07 (59.1 ± 11.7)	0.19 ± 0.39	3.67 ± 0.48	0.02 ± 0.42
	P value	0.0052	0.0022	0.016	0.0001	0.082
P for interaction^a^		0.059	0.26	0.022	0.0022	0.074

Data are expressed as mean ± SD; *P* value refers to simple regression analysis

^#^ *P* < 0.05 vs. RR genotype

^a^Interaction of statin therapy with PON1 genotype

As there was a significant difference in background risk between those using and not using statins (Additional file 1: Table S1), we performed sensitivity analyses using PS. The PS estimated here showed acceptable goodness of fit for the discrimination of patients with and without statin therapy (c statistic: 0.747). The distributions of the PS in the groups using and not using statins almost overlapped (Additional file 3: Figure S1), and the adjustment for and matching with PS could be reliably performed. In the matched design analyses, patients’ characteristics at baseline were almost balanced between the groups with and without statins (Additional file 4: Table S3). All of the three sensitivity analyses showed similar results, with positive linear associations of the number of Q alleles with C-peptide and HOMA2-%β being consistently found across the analyses (Additional file 5: Figure S2, Additional file 6: Figure S3 and Additional file 7: Figure S4).

## Discussion

This is the first study to report the gene–treatment interaction of the *PON1* Q192R polymorphism and statin therapy on insulin secretion among patients with type 2 diabetes. We demonstrated that, when diabetic patients were treated with statins, the number of Q alleles of the *PON1* Q192R polymorphism, the wild-type allele, was associated with increased insulin secretion, while no such association was found among those who were not treated with statins. There were similar favorable influences of the Q allele on fasting plasma glucose and HbA_1c_, although the differences of such effects between the groups with and without statins were not statistically significant.

**Fig. 2:**
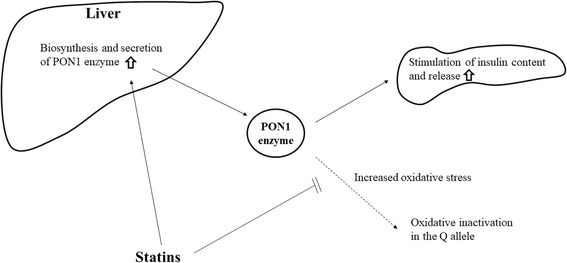
Proposed model for the interaction of PON1 Q192R polymorphism and statins on insulin secretion

The underlying mechanism of how *PON1* Q192R polymorphism and statins interact on insulin secretion in patients with type 2 diabetes remains unclear. One possible explanation is as follows. The PON1 enzyme has the ability to reduce oxidative stress [9] and to increase insulin secretion [10], and the *PON1* Q192R polymorphism has the most significant impact on the enzyme’s activity, with the Q allele being associated with greater enzymatic efficiency [18]. Among patients with type 2 diabetes [19] and with diseases associated with oxidative stress [8], PON1 activity was found to be low, probably due to increased oxidative stress inactivating the PON1 enzyme [20]. Since statins have antioxidant effects and activate the biosynthesis and secretion of the PON1 enzyme in the liver, they may contribute to protecting the PON1 enzyme against inactivation and maintaining its functions [8]. It was also reported that QQ homozygotes showed greater loss of enzyme activity [21] with aging. The enzyme in subjects with this genotype might thus be more vulnerable to such deterioration than that in those with other genotypes and the use of statins could confer their protective effects in this genotype. This possible mechanism is illustrated in Fig. 2. Statins may increase the biosynthesis and secretion of the PON1 enzyme in the liver, and protect the PON1 enzyme from being inactivated by increased oxidative stress. Consequently, insulin secretion may increase in the Q allele carriers.

Some limitations of this study are a lack of the genotyping of other polymorphisms of the *PON1* gene and the lack of availability of data on the serum concentrations and activities of PON1. However, among the major *PON1* polymorphisms, only the Q192R polymorphism has been reported to determine the antioxidant potential of PON1 [9], so the influence of this limitation might be small.

## Conclusions

To the best of our knowledge, this is the first study to investigate the possible glucose-lowering effects of *PON1* Q192R genotypes associated with statin therapy. These findings could partly delineate the complex influence of statins on glucose metabolism. We suggest that *PON1* Q192R genotyping could identify individuals who would benefit from statin therapy for both lipid- and glucose-lowering. Research considering other types of polymorphism in the *PON1* gene, and serum concentrations and activities of PON1 would be helpful in pursuit of the mutual effects of statins and *PON1* polymorphism on glucose metabolism.

## Additional files

Additional file 1: Table S1. Clinical characteristics according to statin therapy. Data are expressed as mean ± SD, median (interquartile), and n (percentage). CVD: cardiovascular disease, OHA: oral hypoglycemic agents, α-GI: alpha-glucosidase inhibitor, DPP4-I: inhibitors of type 4 dipeptidyl peptidase.* log-transformed for the statistical analysis. (DOCX 15 kb)

Additional file 2: Table S2. Clinical characteristics according to PON1 genotype. Data are expressed as mean ± SD, median (interquartile), and n (percentage). CVD: cardiovascular disease, OHA: oral hypoglycemic agents, α-GI: alpha-glucosidase inhibitor, DPP4-I: inhibitors of type 4 dipeptidyl peptidase* log-transformed for the statistical analysis. (DOCX 14 kb)

Additional file 3: Figure S1. Overlap of the distributions of the PS in the groups with and without statins. The band and cross marks inside the boxes represent the median and mean values, respectively. The lower and upper edges of the boxes represent the 25th and 75th percentiles, respectively. The upper and lower lines outside the boxes represent minimum and maximum values. (DOCX 77 kb)

Additional file 4: Table S3. Clinical characteristics according to statin therapy (after 1:1 matching). Data are expressed as mean ± SD, median (interquartile), and n (percentage). CVD: cardiovascular disease, OHA: oral hypoglycemic agents, α-GI: alpha-glucosidase inhibitor, DPP4-I: inhibitors of type 4 dipeptidyl peptidase.* log-transformed for the statistical analysis. (DOCX 14 kb)

Additional file 5: Figure S2. Association of PON1 Q192R polymorphism with FPG, HbA1c, C peptide, and HOMA2-%β after stratified analysis by statin therapy (PS as a covariate). Data are expressed as adjusted mean (95% CI) referring to ANCOVA; *P* value refers to multiple regression analysis. * *P* < 0.05 for trend. (DOCX 67 kb)

Additional file 6: Figure S3. Association of PON1 Q192R polymorphism with FPG, HbA1c, C peptide, and HOMA2-%β after stratified analysis by statin therapy (after 1:1 matching). Data are expressed as mean (SD) referring to ANCOVA; P value refers to multiple regression analysis. * *P* < 0.05 for trend. (DOCX 72 kb)

Additional file 7: Figure S4. Association of PON1 Q192R polymorphism with FPG, HbA1c, C peptide, and HOMA2-%β after stratified analysis by statin therapy (IPTW). Data are expressed as adjusted mean (95% CI) referring to ANCOVA; P value refers to multiple regression analysis. * *P* < 0.05 for trend. (DOCX 68 kb)

## Abbreviations

CVD: Cardiovascular disease
DPP4-I: Inhibitors of type 4 dipeptidyl peptidase
FPG: Fasting plasma glucose
HOMA2-%β: Homeostasis model assessment β-cell function
HOMA2-IR: Homeostasis model assessment insulin resistance
IPTW: Inverse probability of treatment weights
OHA: Oral hypoglycemic agents
PON1: Paraoxonase-1
PS: Propensity scores
SNP: Single-nucleotide polymorphism
α-GI: Alpha-glucosidase inhibitor
