# Supplementary material for: The gene–treatment interaction of paraoxonase-1 gene polymorphism and statin therapy on insulin secretion in Japanese patients with type 2 diabetes: Fukuoka diabetes registry
Source: BMC Med Genet. 2017 Dec 12;18:146. doi: 10.1186/s12881-017-0509-1 (PMC5728066; doi:10.1186/s12881-017-0509-1)
Supplement: Supplementary file 1 — Clinical characteristics according to statin therapy. Data are expressed as mean ± SD, median (interquartile), and n (percentage). CVD: cardiovascular disease, OHA: oral hypoglycemic agents, α-GI: alpha-glucosidase inhibitor, DPP4-I: inhibitors of type 4 dipeptidyl peptidase.* log-transformed for the statistical analysis. (DOCX 15 kb) [file 12881_2017_509_MOESM1_ESM.docx]

Table S1. Clinical characteristics according to statin therapy

|  | Overall | Statin (−) | Statin (+) | P value |
| --- | --- | --- | --- | --- |
|  | N=3798 | N=2120 | N=1678 |  |
| Male, n (%) | 2170 (57.1) | 1388 (65.5) | 782 (46.6) | <0.0001 |
| Age, years | 65.3 ± 10.2 | 64.8 ± 10.7 | 66.1 ± 9.5 | 0.0004 |
| BMI, kg/m^2^ | 24.1 ± 3.8 | 23.9 ± 3.9 | 24.4 ± 3.7 | 0.0001 |
| Duration of diabetes, years | 14.6 ± 10.1 | 14.3 ± 10.3 | 14.9 ± 9.9 | 0.083 |
| Hypertension, n (%) | 2438 (64.2) | 1277 (60.2) | 1161 (69.2) | <0.0001 |
| Current smoker, n (%) | 682 (17.9) | 446 (21.0) | 236 (14.1) | <0.0001 |
| Current drinker, n (%) | 1494 (39.3) | 946 (44.6) | 548 (32.7) | <0.0001 |
| Leisure-time physical activity, METs·h/w | 18.7 ± 18.3 | 18.9 ± 18.4 | 18.4 ± 18.2 | 0.43 |
| Family history of diabetes, n (%) | 2092 (55.1) | 1148 (54.2) | 944 (56.3) | 0.19 |
| Family history of hyperlipidemia, n (%) | 229 (6.0) | 109 (5.1) | 120 (7.2) | 0.0098 |
| Past history of CVD, n (%) | 916 (24.1) | 403 (19.0) | 513 (30.4) | <0.0001 |
| HbA_1c_, % (mmol/mol) | 7.4 ± 1.0 (57.4 ± 10.9 ) | 7.3 ± 1.0 (56.3 ± 10.9 ) | 7.5 ± 1.0 (58.5 ± 10.9 ) | <0.0001 |
| Fasting plasma glucose, mmol/l | 7.68 ± 1.99 | 7.70 ± 2.04 | 7.65 ± 1.92 | 0.39 |
| HDL cholesterol, mmol/l | 1.44 ± 0.37 | 1.42 ± 0.38 | 1.46 ± 0.37 | 0.0014 |
| LDL cholesterol, mmol/l | 2.88 ± 0.70 | 3.02 ± 0.73 | 2.70 ± 0.62 | <0.0001 |
| Triglyceride^*^, mmol/l | 1.23 (0.91, 1.73) | 1.20 (0.90, 1.72) | 1.29 (0.93, 1.74) | 0.043 |
| Method for controlling blood glucose: diet, OHA, insulin, combination of OHA and insulin, n (%), respectively | 738 (19.4), 2287 (60.2), 495 (13.0), 278 (7.3) | 447 (21.1), 1238 (58.4), 297 (14.0), 138 (6.5) | 291 (17.3), 1049 (62.5), 198 (11.8), 140 (8.3) | 0.0006 |
| OHA, n (%) | 2565 (67.5) | 1376 (64.9) | 1189 (70.9) | <0.0001 |
| Sulfonylurea, n (%) | 1740 (45.8) | 911 (43.0) | 829 (49.4) | <0.0001 |
| Biguanide, n (%) | 1357 (35.7) | 706 (33.3) | 651 (38.8) | 0.0004 |
| α-GI, n (%) | 453 (11.9) | 228 (10.8) | 225 (13.4) | 0.012 |
| Thiazolidine, n (%) | 541 (14.2) | 245 (11.6) | 296 (17.6) | <0.0001 |
| Glinide, n (%) | 229 (6.0) | 134 (6.32) | 95 (5.7) | 0.40 |
| DPP4-I, n (%) | 15 (0.4) | 10 (0.47) | 5 (0.3) | 0.40 |
| Insulin, n (%) | 773 (20.3) | 435 (20.5) | 338 (20.1) | 0.78 |
| Antiplatelet, n (%) | 955 (25.1) | 398 (18.8) | 557 (33.2) | <0.0001 |
| Fibrate, n (%) | 159 (4.2) | 133 (6.3) | 26 (1.55) | <0.0001 |
| Ezetimibe, n (%) | 68 (1.8) | 33 (1.6) | 35 (2.1) | 0.22 |
| Ethyl eicosapentate, n (%) | 82 (2.2) | 35 (1.7) | 47 (2.8) | 0.016 |

Data are expressed as mean ± SD, median (interquartile), and n (percentage). CVD: cardiovascular disease, OHA: oral hypoglycemic agents, α-GI: alpha-glucosidase inhibitor, DPP4-I: inhibitors of type 4 dipeptidyl peptidase

* log-transformed for the statistical analysis.
