# Supplementary material for: The gene–treatment interaction of paraoxonase-1 gene polymorphism and statin therapy on insulin secretion in Japanese patients with type 2 diabetes: Fukuoka diabetes registry
Source: BMC Med Genet. 2017 Dec 12;18:146. doi: 10.1186/s12881-017-0509-1 (PMC5728066; doi:10.1186/s12881-017-0509-1)
Supplement: Supplementary file 2 — Clinical characteristics according to PON1 genotype. Data are expressed as mean ± SD, median (interquartile), and n (percentage). CVD: cardiovascular disease, OHA: oral hypoglycemic agents, α-GI: alpha-glucosidase inhibitor, DPP4-I: inhibitors of type 4 dipeptidyl peptidase* log-transformed for the statistical analysis. (DOCX 14 kb) [file 12881_2017_509_MOESM2_ESM.docx]

Table S2. Clinical characteristics according to PON1 genotype

|  |  | Overall |  |  |
| --- | --- | --- | --- | --- |
| PON1 Q192R genotype | QQ | QR | RR | P value |
|  | N=428, 11.2% | N=1654, 43.6% | N=1716, 45.2% |  |
| Male, n (%) | 245 (57.2) | 944 (57.1) | 981 (57.2) | 0.99 |
| Age, years | 65.6 ± 10.2 | 65.4 ± 10.2 | 65.2 ± 10.2 | 0.78 |
| BMI, kg/m^2^ | 24.1 ± 3.8 | 24.0 ± 3.9 | 24.2 ± 3.7 | 0.48 |
| Duration of diabetes, years | 14.0 ± 10.5 | 14.5 ± 10.0 | 14.8 ± 10.2 | 0.26 |
| Current smoker, n (%) | 79 (18.5) | 278 (16.8) | 325 (18.9) | 0.26 |
| Current drinker, n (%) | 175 (40.9) | 642 (38.8) | 677 (39.5) | 0.73 |
| Leisure-time physical activity, METs·h/w | 17.8 ± 18.0 | 18.8 ± 18.4 | 18.7 ± 18.2 | 0.58 |
| Family history of diabetes, n (%) | 230 (53.7) | 937 (56.7) | 925 (53.9) | 0.23 |
| Past history of CVD, n (%) | 105 (24.5) | 401 (24.2) | 410 (23.9) | 0.96 |
| HDL cholesterol, mmol/l | 1.45 ± 0.37 | 1.45 ± 0.38 | 1.43 ± 0.37 | 0.41 |
| LDL cholesterol, mmol/l | 2.89 ± 0.73 | 2.88 ± 0.69 | 2.88 ± 0.71 | 0.93 |
| OHA, n (%) | 268 (62.6) | 1127 (68.1) | 1170 (68.2) | 0.070 |
| Sulfonylurea, n (%) | 168(39.3) | 787(47.6) | 785(45.8) | 0.009 |
| Biguanide, n (%) | 142(33.2) | 578(35.0) | 637(37.1) | 0.21 |
| α-GI, n (%) | 57(13.3) | 214(12.9) | 182(10.6) | 0.07 |
| Thiazolidine, n (%) | 56(13.1) | 259(15.7) | 226(13.2) | 0.09 |
| Glinide, n (%) | 23(5.4) | 107(6.5) | 99(5.8) | 0.58 |
| DPP4-I, n (%) | 1(0.23) | 6(0.36) | 8(0.47) | 0.93 |
| Insulin, n (%) | 96(22.4) | 323(19.5) | 354(20.6) | 0.38 |

Data are expressed as mean ± SD, median (interquartile), and n (percentage). CVD: cardiovascular disease, OHA: oral hypoglycemic agents, α-GI: alpha-glucosidase inhibitor, DPP4-I: inhibitors of type 4 dipeptidyl peptidase

* log-transformed for the statistical analysis.
