# Supplementary figures and images for: The gene–treatment interaction of paraoxonase-1 gene polymorphism and statin therapy on insulin secretion in Japanese patients with type 2 diabetes: Fukuoka diabetes registry
Source: BMC Med Genet. 2017 Dec 12;18:146. doi: 10.1186/s12881-017-0509-1 (PMC5728066; doi:10.1186/s12881-017-0509-1)

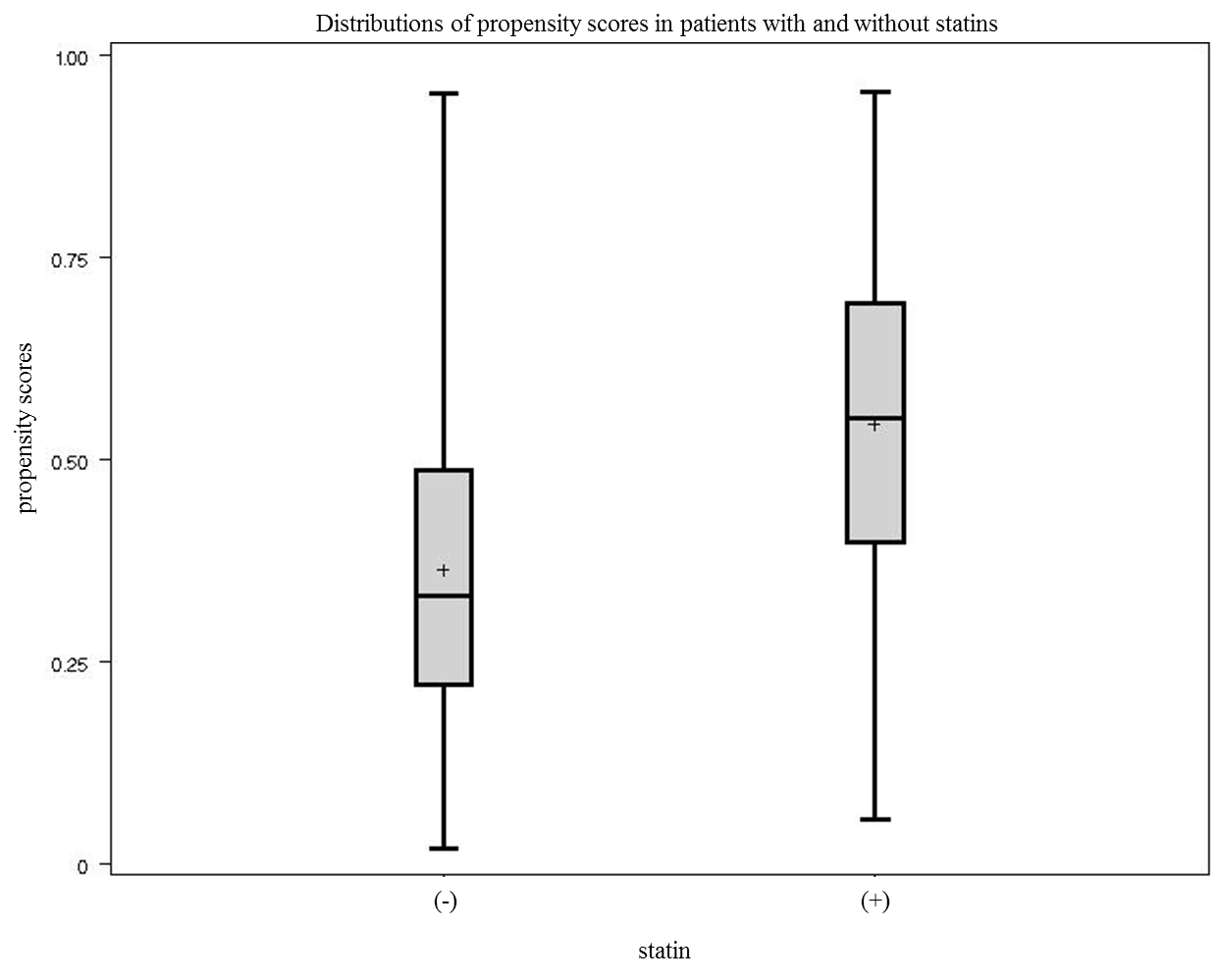


Figure S1.

Supplement: Supplementary file 3 — Overlap of the distributions of the PS in the groups with and without statins. The band and cross marks inside the boxes represent the median and mean values, respectively. The lower and upper edges of the boxes represent the 25th and 75th percentiles, respectively. The upper and lower lines outside the boxes represent minimum and maximum values. (DOCX 77 kb) [file 12881_2017_509_MOESM3_ESM.docx]

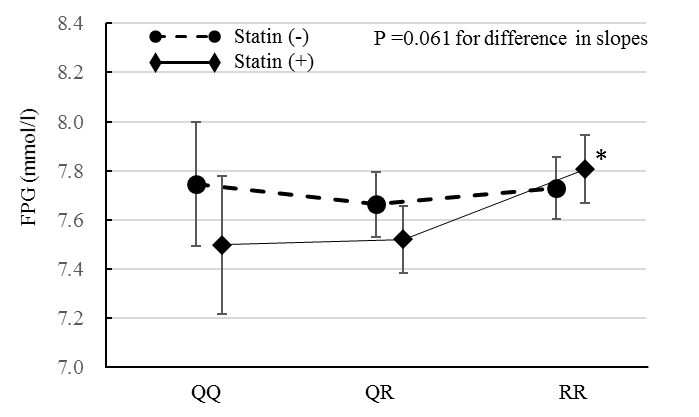

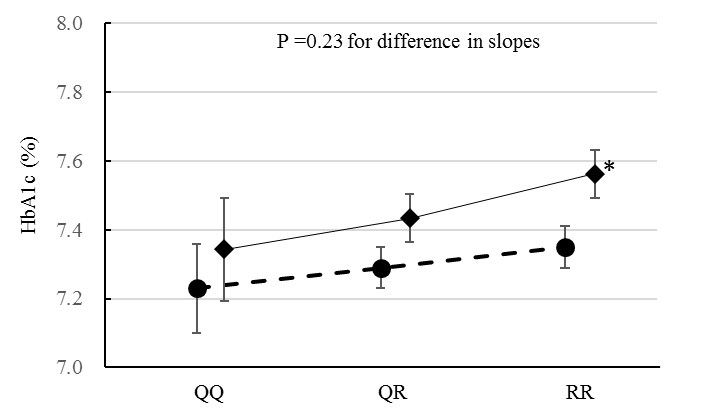


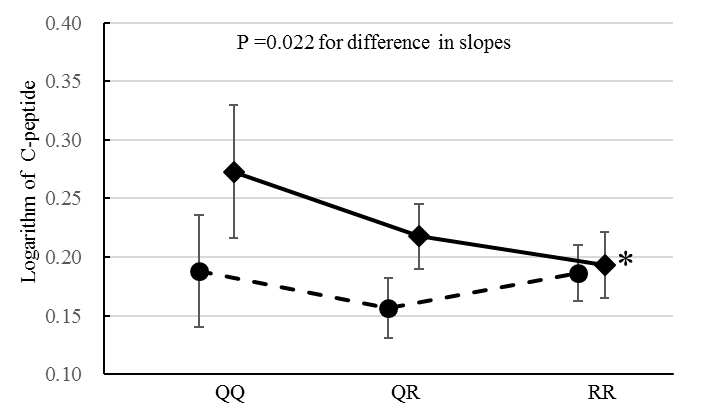

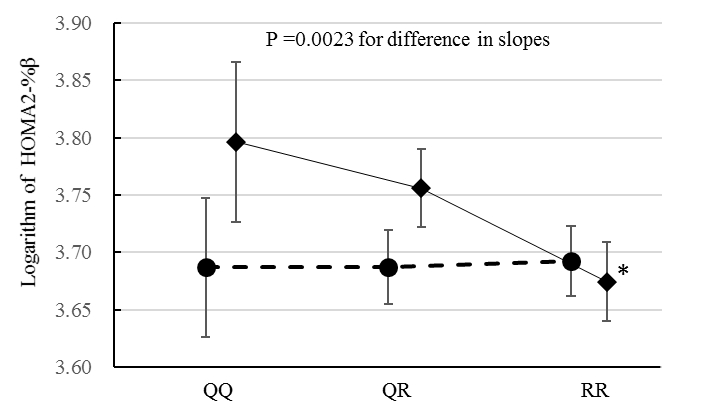


Figure S2.

Supplement: Supplementary file 5 — Association of PON1 Q192R polymorphism with FPG, HbA1c, C peptide, and HOMA2-%β after stratified analysis by statin therapy (PS as a covariate). Data are expressed as adjusted mean (95% CI) referring to ANCOVA; P value refers to multiple regression analysis. * P < 0.05 for trend. (DOCX 67 kb) [file 12881_2017_509_MOESM5_ESM.docx]

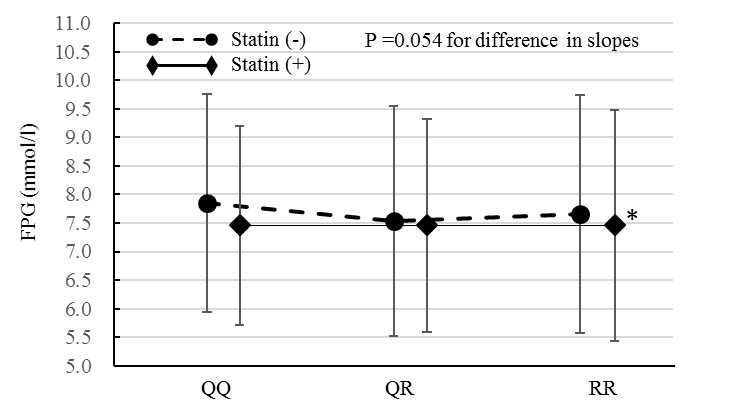

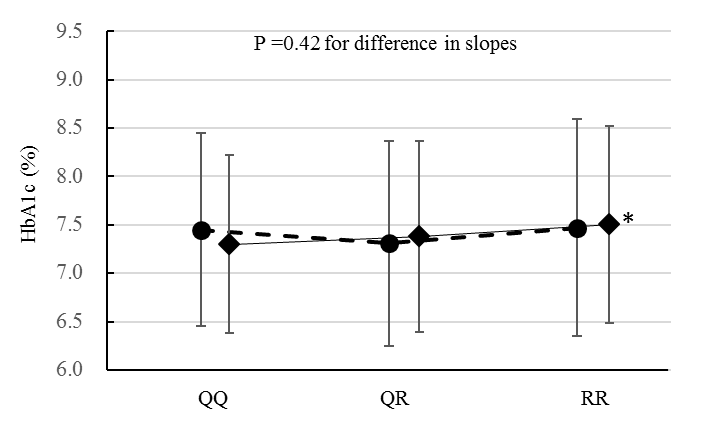

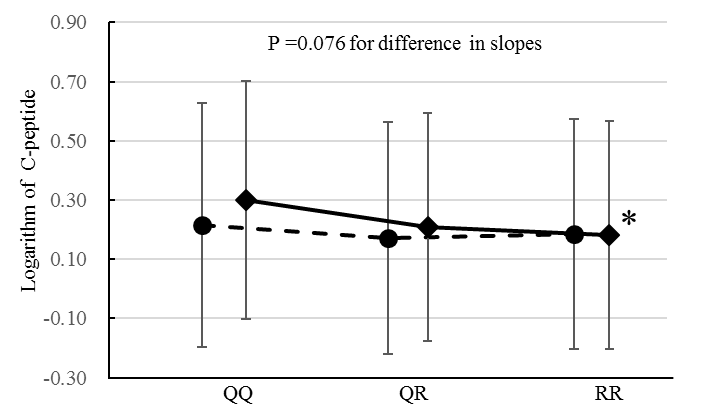

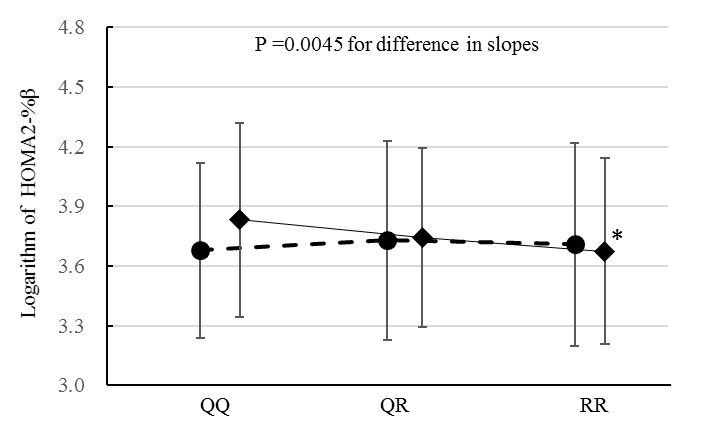


Figure S3.

Supplement: Supplementary file 6 — Association of PON1 Q192R polymorphism with FPG, HbA1c, C peptide, and HOMA2-%β after stratified analysis by statin therapy (after 1:1 matching). Data are expressed as mean (SD) referring to ANCOVA; P value refers to multiple regression analysis. * P < 0.05 for trend. (DOCX 72 kb) [file 12881_2017_509_MOESM6_ESM.docx]

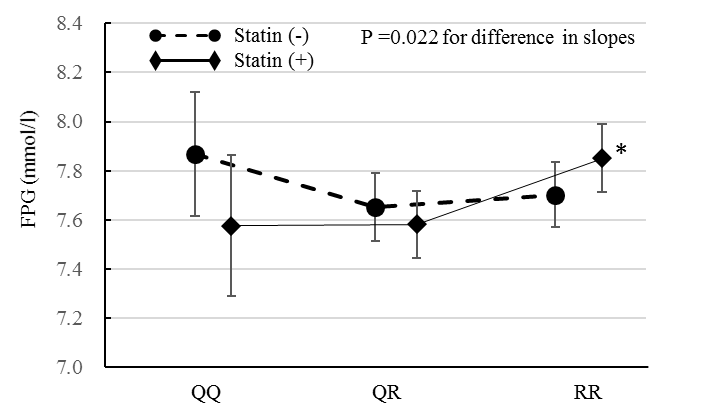

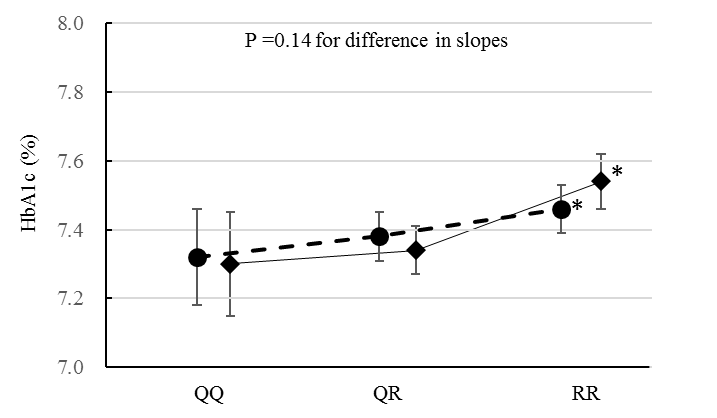


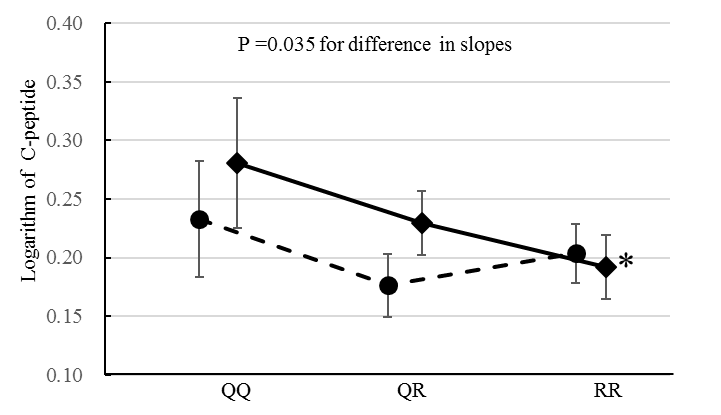

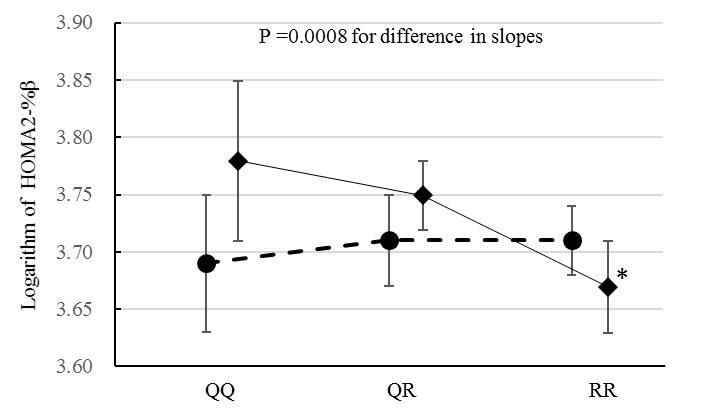


Figure S4.

Supplement: Supplementary file 7 — Association of PON1 Q192R polymorphism with FPG, HbA1c, C peptide, and HOMA2-%β after stratified analysis by statin therapy (IPTW). Data are expressed as adjusted mean (95% CI) referring to ANCOVA; P value refers to multiple regression analysis. * P < 0.05 for trend. (DOCX 68 kb) [file 12881_2017_509_MOESM7_ESM.docx]
