# Supplementary material for: The gene–treatment interaction of paraoxonase-1 gene polymorphism and statin therapy on insulin secretion in Japanese patients with type 2 diabetes: Fukuoka diabetes registry
Source: BMC Med Genet. 2017 Dec 12;18:146. doi: 10.1186/s12881-017-0509-1 (PMC5728066; doi:10.1186/s12881-017-0509-1)
Supplement: Supplementary file 4 — Clinical characteristics according to statin therapy (after 1:1 matching). Data are expressed as mean ± SD, median (interquartile), and n (percentage). CVD: cardiovascular disease, OHA: oral hypoglycemic agents, α-GI: alpha-glucosidase inhibitor, DPP4-I: inhibitors of type 4 dipeptidyl peptidase.* log-transformed for the statistical analysis. (DOCX 14 kb) [file 12881_2017_509_MOESM4_ESM.docx]

Table S3. Clinical characteristics according to statin therapy (after 1:1 matching)

|  | Statin (−) | Statin (+) | P value |
| --- | --- | --- | --- |
|  | N=1205 | N=1205 |  |
| Male, n (%) | 653 (54.2) | 647 (53.7) | 0.81 |
| Age, years | 65.4 ± 10.6 | 65.4 ± 9.7 | 0.94 |
| BMI, kg/m^2^ | 23.9 ± 3.9 | 24.1 ± 3.5 | 0.78 |
| Duration of diabetes, years | 14.9 ± 10.3 | 15.0 ± 10.1 | 0.81 |
| Hypertension, n (%) | 794 (65.9) | 789 (65.9) | 0.83 |
| Current smoker, n (%) | 217 (18.0) | 190 (15.8) | 0.14 |
| Current drinker, n (%) | 495 (41.1) | 425 (35.3) | 0.0033 |
| Leisure-time physical activity, METs·h/w | 18.8 ± 18.6 | 18.7 ± 18.3 | 0.83 |
| Family history of diabetes, n (%) | 653 (54.2) | 666 (55.3) | 0.59 |
| Family history of hyperlipidemia, n (%) | 68 (5.6) | 69 (5.7) | 0.93 |
| Past history of CVD, n (%) | 291 (24.2) | 294 (24.4) | 0.89 |
| HbA1c, % (mmol/mol) | 7.4 ± 1.1 (57.4 ± 12.0 ) | 7.4 ± 1.0 (57.4 ± 10.9) | 0.97 |
| Fasting plasma glucose, mmol/l | 7.63 ± 2.03 | 7.67 ± 1.93 | 0.61 |
| HDL cholesterol, mmol/l | 1.45 ± 0.41 | 1.46 ± 0.37 | 0.47 |
| LDL cholesterol, mmol/l | 2.82 ± 0.71 | 2.82 ± 0.62 | 0.96 |
| Triglyceride^*^, mmol/l | 1.22 (0.88, 1.80) | 1.28 (0.91, 1.75) | 0.83 |
| Method for controlling blood glucose: diet, OHA, insulin, combination of OHA and insulin, n (%), respectively | 223 (18.5), 739(61.3), 152 (12.6), 91 (7.6) | 232 (19.3), 730 (60.6), 138 (11.5), 105 (8.7) | 0.59 |
| OHA, n (%) | 830 (68.9) | 835 (69.3) | 0.83 |
| Sulfonylurea, n (%) | 563 (46.7) | 572 (47.5) | 0.71 |
| Biguanide, n (%) | 440 (36.5) | 451 (37.4) | 0.64 |
| α-GI, n (%) | 151 (12.5) | 144 (12.0) | 0.66 |
| Thiazolidine, n (%) | 173 (14.4) | 175 (14.5) | 0.91 |
| Glinide, n (%) | 70 (5.8) | 72 (6.0) | 0.86 |
| DPP4-I, n (%) | 4 (0.33) | 4 (0.33) | 1.0 |
| Insulin, n (%) | 243 (20.2) | 243 (20.2) | 1.0 |
| Antiplatelet, n (%) | 299 (24.8) | 302 (25.1) | 0.89 |
| Fibrate, n (%) | 28 (2.3) | 25 (2.1) | 0.68 |
| Ezetimibe, n (%) | 24 (2.0) | 18 (1.5) | 0.35 |
| Ethyl eicosapentate, n (%) | 26 (2.2) | 27 (2.2) | 0.89 |

Data are expressed as mean ± SD, median (interquartile), and n (percentage). CVD: cardiovascular disease, OHA: oral hypoglycemic agents, α-GI: alpha-glucosidase inhibitor, DPP4-I: inhibitors of type 4 dipeptidyl peptidase

* log-transformed for the statistical analysis.
